# (1R*,2R*)-1-(4-Chloro­phen­yl)-4-dimethyl­amino-1-(3-meth­oxy-2-naphth­yl)-2-(1-naphth­yl)butan-2-ol

**DOI:** 10.1107/S160053681001891X

**Published:** 2010-06-05

**Authors:** Ping Liu, Junhai Xiao, Wu Zhong, Song Li, Xiaohong Yang

**Affiliations:** a Department of Medicinal Chemistry, School of Pharmacy, Jilin University, Changchun 130021, People’s Republic of China; b Beijing Institute of Pharmacology and Toxicology, Beijing 100850, People’s Republic of China

## Abstract

In the title compound, C_33_H_32_ClNO_2_, the benzene ring is oriented at dihedral angles of 6.23 (5) and 66.44 (5)° with respect to the two naphthalene ring systems. An intra­molecular O—H⋯N hydrogen bond between the hydr­oxy H atom and the amine N atom generates an *S*(6) ring.

## Related literature

For general background and the synthesis of diaryl­quinoline anti-tuberculosis drugs, see: Cohen (2004 ▶), Andries *et al.* (2005 ▶); Guillemont *et al.* (2004 ▶).
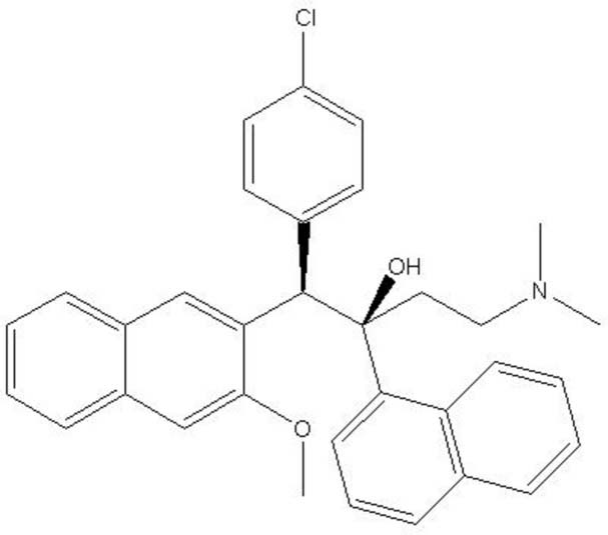


## Experimental

### 

#### Crystal data

C_33_H_32_ClNO_2_
*M* *_r_* = 510.05
Monoclinic, 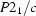

*a* = 18.712 (5) Å
*b* = 9.135 (2) Å
*c* = 16.369 (4) Å
β = 111.991 (4)°
*V* = 2594.2 (11) Å^3^
*Z* = 4
Mo *K*α radiation
μ = 0.18 mm^−1^
*T* = 116 K
0.20 × 0.16 × 0.12 mm

#### Data collection

Rigaku Saturn CCD diffractometer
Absorption correction: multi-scan (*CrystalClear*; Rigaku/MSC, 2005 ▶) *T*_min_ = 0.965, *T*_max_ = 0.979
18901 measured reflections
4573 independent reflections
3918 reflections with *I* > 2σ(*I*)
*R*_int_ = 0.032

#### Refinement

*R*[*F* ^2^ > 2σ(*F* ^2^)] = 0.036
*wR*(*F* ^2^) = 0.099
*S* = 1.09
4573 reflections
338 parameters
H-atom parameters constrained
Δρ_max_ = 0.18 e Å^−3^
Δρ_min_ = −0.31 e Å^−3^

### 

Data collection: *CrystalClear* (Rigaku/MSC, 2005 ▶); cell refinement: *CrystalClear*; data reduction: *CrystalClear*; program(s) used to solve structure: *SHELXS97* (Sheldrick, 2008 ▶); program(s) used to refine structure: *SHELXL97* (Sheldrick, 2008 ▶); molecular graphics: *SHELXTL* (Sheldrick, 2008 ▶); software used to prepare material for publication: *CrystalStructure* (Rigaku/MSC, 2005 ▶).

## Supplementary Material

Crystal structure: contains datablocks I, global. DOI: 10.1107/S160053681001891X/hb5430sup1.cif

Structure factors: contains datablocks I. DOI: 10.1107/S160053681001891X/hb5430Isup2.hkl

Additional supplementary materials:  crystallographic information; 3D view; checkCIF report

## Figures and Tables

**Table 1:** Hydrogen-bond geometry (Å, °)

*D*—H⋯*A*	*D*—H	H⋯*A*	*D*⋯*A*	*D*—H⋯*A*
O2—H2⋯N1	0.82	1.93	2.6995 (17)	157

**Fig. 1.**
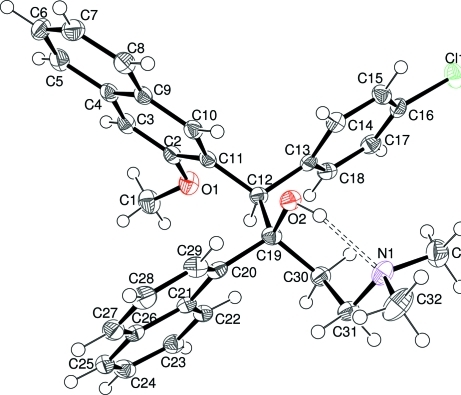
Ellipsoid plot

## supplementary crystallographic information

### Comment

The compound (1*R*,2S)-1-(6-bromo-2-methoxyquinolin-3-yl)-4-(dimethylamino)-2-(naphthalene- 1-yl)-1-phenylbutan-2-ol, is a promising drug against tuberculosis (Andries *et al.*, 2005; Cohen, 2004 and Guillemont  *et al.* 2004). We modified this compound in order to get some more efficient antituberculosis drugs. To characterize our product its single crystal structure was determined.

In the molecule of the title compound (Fig.1), the dihedral angle between the naphthalene ring (C20—C29) and the benzene ring (C13—C18) amount to 6.232 (46)° whereas the other naphthalene ring (C2—C10) is oriented with respect to the benzene ring at a dihedral angle of 66.438 (51)°. In the structure an intramolecular O—H···N hydrogen bond is found (Tab. 1).

### Experimental

n-BuLi (2.5M in hexanes, 4 ml, 10 mmol) was added slowly at 233 K under N_2_ to a solution of diisopropylamine (1.4 ml, 10 mmol) in THF (15 ml). The mixture was stirred at 233k for 30 min, then cooled to 195 K. Afterwards a solution of 2-(4-chlorobenzyl)-3-methoxynaphthalene (2.59 g, 9.2 mmol) in THF (20 ml) was added slowly. The mixture was stirred at 195 K for about 40 min and then a solution of 3-(dimethylamino)-1-(naphthalen-1-yl)propan-1-one (2.9 g, 12.8 mmol) in THF (20 ml) was added slowly. The mixture was stirred at 195 K for 8 h, hydrolyzed with ice water at 233 K and extracted with ethyl acetate. The organic layer was separated, dried over MgSO_4_, filtered and the solvent was evaporated. The residue was purified by column chromatography over silica gel (eluent: petroleum ether/ethyl acetate, 50/1). Two fractions were collected (Guillemont *et al.*, 2004). On evaporation of the solvent (petroleum ether/ethyl acetate, 50/1) from fraction at room temperature in air colourless prisms of (I) were obtained.

### Refinement

All H atoms were positioned with ideal geometry (O—H H atoms allowed to rotate but not to tip) and with d(C—H)=0.93 Å for aromatic, 0.98 Å for CH, 0.97 Å for CH_2_ and 0.96 Å for CH_3 _atoms and were refined with Uiso(H) = 1.2 Ueq(C) for CH and CH_2_ H atoms and Uiso(H) = 1.5 Ueq(C) for CH_3 _and O—H H atoms.

### Crystal data

**Table:**

C_33_H_32_ClNO_2_	*F*(000) = 1080
*M**_r_* = 510.05	*D*_x_ = 1.306 Mg m^−^^3^
Monoclinic, *P*2_1_/*c*	Mo *K*α radiation, λ = 0.71073 Å
Hall symbol: -P 2ybc	Cell parameters from 8940 reflections
*a* = 18.712 (5) Å	θ = 2.2–27.9°
*b* = 9.135 (2) Å	µ = 0.18 mm^−^^1^
*c* = 16.369 (4) Å	*T* = 116 K
β = 111.991 (4)°	Prism, colorless
*V* = 2594.2 (11) Å^3^	0.20 × 0.16 × 0.12 mm
*Z* = 4	

### Data collection

**Table:**

Rigaku Saturn CCD diffractometer	4573 independent reflections
Radiation source: rotating anode	3918 reflections with *I* > 2σ(*I*)
multilayer	*R*_int_ = 0.032
Detector resolution: 14.63 pixels mm^-1^	θ_max_ = 25.0°, θ_min_ = 2.4°
ω and φ scans	*h* = −19→22
Absorption correction: multi-scan (*CrystalClear*; Rigaku/MSC, 2005)	*k* = −10→10
*T*_min_ = 0.965, *T*_max_ = 0.979	*l* = −19→19
18901 measured reflections	

### Refinement

**Table:**

Refinement on *F*^2^	Primary atom site location: structure-invariant direct methods
Least-squares matrix: full	Secondary atom site location: difference Fourier map
*R*[*F*^2^ > 2σ(*F*^2^)] = 0.036	Hydrogen site location: inferred from neighbouring sites
*wR*(*F*^2^) = 0.099	H-atom parameters constrained
*S* = 1.09	*w* = 1/[σ^2^(*F*_o_^2^) + (0.0587*P*)^2^ + 0.0772*P*] where *P* = (*F*_o_^2^ + 2*F*_c_^2^)/3
4573 reflections	(Δ/σ)_max_ = 0.001
338 parameters	Δρ_max_ = 0.18 e Å^−^^3^
0 restraints	Δρ_min_ = −0.31 e Å^−^^3^

### Special details

**Table:**

Geometry. All esds (except the esd in the dihedral angle between two l.s. planes) are estimated using the full covariance matrix. The cell esds are taken into account individually in the estimation of esds in distances, angles and torsion angles; correlations between esds in cell parameters are only used when they are defined by crystal symmetry. An approximate (isotropic) treatment of cell esds is used for estimating esds involving l.s. planes.
Refinement. Refinement of *F*^2^ against ALL reflections. The weighted *R*-factor wR and goodness of fit *S* are based on *F*^2^, conventional *R*-factors *R* are based on *F*, with *F* set to zero for negative *F*^2^. The threshold expression of *F*^2^ > σ(*F*^2^) is used only for calculating *R*-factors(gt) etc. and is not relevant to the choice of reflections for refinement. *R*-factors based on *F*^2^ are statistically about twice as large as those based on *F*, and *R*- factors based on ALL data will be even larger.

### Fractional atomic coordinates and isotropic or equivalent isotropic displacement parameters (Å^2^)

**Table:**

	*x*	*y*	*z*	*U*_iso_*/*U*_eq_	
Cl1	0.08594 (2)	−0.07359 (5)	0.46621 (2)	0.03327 (14)	
O1	0.37601 (6)	0.47214 (13)	0.67039 (6)	0.0280 (3)	
O2	0.15042 (6)	0.45626 (11)	0.76790 (6)	0.0224 (2)	
H2	0.1037	0.4674	0.7520	0.034*	
N1	0.00317 (7)	0.54248 (15)	0.68199 (8)	0.0262 (3)	
C1	0.43816 (10)	0.55500 (19)	0.66354 (11)	0.0350 (4)	
H1A	0.4497	0.6357	0.7040	0.053*	
H1B	0.4238	0.5914	0.6045	0.053*	
H1C	0.4828	0.4936	0.6775	0.053*	
C2	0.38276 (8)	0.42853 (16)	0.75343 (9)	0.0213 (3)	
C3	0.45183 (8)	0.39820 (17)	0.81939 (9)	0.0234 (3)	
H3	0.4971	0.4067	0.8088	0.028*	
C4	0.45519 (8)	0.35391 (16)	0.90395 (9)	0.0220 (3)	
C5	0.52573 (9)	0.32564 (17)	0.97478 (10)	0.0269 (4)	
H5	0.5719	0.3335	0.9660	0.032*	
C6	0.52683 (9)	0.28711 (18)	1.05574 (10)	0.0294 (4)	
H6	0.5736	0.2686	1.1015	0.035*	
C7	0.45778 (9)	0.27531 (17)	1.07038 (10)	0.0287 (4)	
H7	0.4590	0.2499	1.1259	0.034*	
C8	0.38864 (9)	0.30102 (17)	1.00330 (10)	0.0252 (4)	
H8	0.3432	0.2918	1.0136	0.030*	
C9	0.38510 (8)	0.34155 (16)	0.91827 (9)	0.0212 (3)	
C10	0.31462 (8)	0.37265 (16)	0.84820 (9)	0.0211 (3)	
H10	0.2688	0.3636	0.8577	0.025*	
C11	0.31159 (8)	0.41579 (15)	0.76655 (9)	0.0192 (3)	
C12	0.23748 (8)	0.45229 (16)	0.69006 (9)	0.0191 (3)	
H12	0.2530	0.5143	0.6507	0.023*	
C13	0.20123 (8)	0.31600 (16)	0.63590 (9)	0.0190 (3)	
C14	0.17019 (8)	0.20240 (17)	0.66875 (9)	0.0222 (3)	
H14	0.1730	0.2070	0.7266	0.027*	
C15	0.13521 (8)	0.08287 (17)	0.61745 (9)	0.0237 (3)	
H15	0.1140	0.0087	0.6402	0.028*	
C16	0.13221 (8)	0.07529 (17)	0.53164 (9)	0.0227 (3)	
C17	0.16557 (9)	0.18162 (17)	0.49810 (9)	0.0250 (4)	
H17	0.1655	0.1735	0.4414	0.030*	
C18	0.19958 (8)	0.30198 (16)	0.55077 (9)	0.0225 (3)	
H18	0.2217	0.3748	0.5282	0.027*	
C19	0.17914 (8)	0.54677 (16)	0.71574 (9)	0.0205 (3)	
C20	0.21941 (8)	0.68257 (16)	0.76984 (9)	0.0208 (3)	
C21	0.26013 (8)	0.79135 (16)	0.73961 (9)	0.0209 (3)	
C22	0.26584 (8)	0.79312 (16)	0.65518 (9)	0.0214 (3)	
H22	0.2414	0.7199	0.6149	0.026*	
C23	0.30608 (8)	0.89908 (17)	0.63137 (10)	0.0247 (4)	
H23	0.3079	0.8969	0.5754	0.030*	
C24	0.34450 (9)	1.01083 (17)	0.69010 (11)	0.0281 (4)	
H24	0.3725	1.0813	0.6738	0.034*	
C25	0.34029 (8)	1.01492 (18)	0.77106 (10)	0.0271 (4)	
H25	0.3658	1.0891	0.8100	0.033*	
C26	0.29797 (8)	0.90910 (17)	0.79797 (9)	0.0218 (3)	
C27	0.29246 (9)	0.92087 (17)	0.88158 (10)	0.0278 (4)	
H27	0.3165	0.9979	0.9189	0.033*	
C28	0.25203 (9)	0.81963 (18)	0.90722 (10)	0.0293 (4)	
H28	0.2475	0.8289	0.9617	0.035*	
C29	0.21699 (9)	0.70118 (17)	0.85237 (9)	0.0249 (4)	
H29	0.1910	0.6321	0.8725	0.030*	
C30	0.11066 (8)	0.59297 (17)	0.63159 (9)	0.0227 (3)	
H30A	0.1286	0.6659	0.6006	0.027*	
H30B	0.0936	0.5085	0.5933	0.027*	
C31	0.04188 (9)	0.65525 (17)	0.64869 (10)	0.0265 (4)	
H31A	0.0593	0.7339	0.6914	0.032*	
H31B	0.0053	0.6959	0.5944	0.032*	
C32	−0.04747 (10)	0.6089 (2)	0.72092 (11)	0.0421 (5)	
H32A	−0.0868	0.6645	0.6769	0.063*	
H32B	−0.0180	0.6725	0.7682	0.063*	
H32C	−0.0710	0.5336	0.7433	0.063*	
C33	−0.04136 (10)	0.44129 (19)	0.61219 (11)	0.0365 (4)	
H33A	−0.0670	0.3714	0.6355	0.055*	
H33B	−0.0073	0.3910	0.5900	0.055*	
H33C	−0.0789	0.4951	0.5653	0.055*	

### Atomic displacement parameters (Å^2^)

**Table:**

	*U*^11^	*U*^22^	*U*^33^	*U*^12^	*U*^13^	*U*^23^
Cl1	0.0334 (2)	0.0323 (3)	0.0330 (2)	−0.00749 (18)	0.01111 (19)	−0.01273 (17)
O1	0.0230 (6)	0.0368 (7)	0.0278 (6)	−0.0038 (5)	0.0136 (5)	0.0018 (5)
O2	0.0187 (5)	0.0247 (6)	0.0265 (5)	0.0016 (5)	0.0115 (5)	0.0046 (4)
N1	0.0195 (7)	0.0352 (8)	0.0249 (7)	0.0038 (6)	0.0096 (6)	0.0013 (6)
C1	0.0330 (10)	0.0371 (11)	0.0395 (9)	−0.0092 (8)	0.0187 (8)	0.0022 (8)
C2	0.0217 (8)	0.0184 (9)	0.0253 (8)	−0.0019 (6)	0.0105 (7)	−0.0033 (6)
C3	0.0173 (8)	0.0223 (9)	0.0327 (8)	−0.0017 (6)	0.0119 (7)	−0.0057 (7)
C4	0.0217 (8)	0.0162 (8)	0.0275 (8)	0.0002 (6)	0.0085 (7)	−0.0060 (6)
C5	0.0209 (8)	0.0237 (9)	0.0357 (9)	0.0008 (7)	0.0101 (7)	−0.0085 (7)
C6	0.0241 (9)	0.0263 (10)	0.0287 (8)	0.0061 (7)	−0.0006 (7)	−0.0041 (7)
C7	0.0333 (9)	0.0247 (9)	0.0253 (8)	0.0036 (7)	0.0077 (7)	0.0000 (7)
C8	0.0251 (8)	0.0233 (9)	0.0281 (8)	−0.0012 (7)	0.0109 (7)	−0.0013 (7)
C9	0.0215 (8)	0.0157 (8)	0.0254 (8)	−0.0008 (6)	0.0076 (7)	−0.0036 (6)
C10	0.0187 (8)	0.0185 (8)	0.0266 (8)	−0.0007 (6)	0.0092 (7)	−0.0013 (6)
C11	0.0185 (8)	0.0147 (8)	0.0248 (7)	−0.0008 (6)	0.0086 (6)	−0.0036 (6)
C12	0.0183 (8)	0.0189 (8)	0.0219 (7)	−0.0003 (6)	0.0094 (6)	0.0019 (6)
C13	0.0142 (7)	0.0201 (8)	0.0217 (7)	0.0034 (6)	0.0057 (6)	0.0015 (6)
C14	0.0213 (8)	0.0248 (9)	0.0213 (7)	0.0007 (6)	0.0090 (6)	−0.0001 (6)
C15	0.0218 (8)	0.0218 (9)	0.0299 (8)	−0.0020 (6)	0.0127 (7)	0.0004 (6)
C16	0.0167 (8)	0.0240 (9)	0.0238 (7)	0.0025 (6)	0.0034 (6)	−0.0049 (6)
C17	0.0265 (9)	0.0279 (9)	0.0195 (7)	0.0046 (7)	0.0072 (7)	0.0009 (6)
C18	0.0225 (8)	0.0213 (9)	0.0251 (8)	0.0030 (6)	0.0104 (7)	0.0043 (6)
C19	0.0214 (8)	0.0196 (8)	0.0231 (7)	0.0004 (6)	0.0111 (6)	0.0028 (6)
C20	0.0187 (8)	0.0212 (9)	0.0220 (7)	0.0065 (6)	0.0071 (6)	0.0023 (6)
C21	0.0186 (8)	0.0187 (8)	0.0243 (7)	0.0052 (6)	0.0069 (6)	0.0016 (6)
C22	0.0217 (8)	0.0187 (9)	0.0240 (7)	0.0034 (6)	0.0088 (6)	0.0002 (6)
C23	0.0230 (8)	0.0249 (9)	0.0282 (8)	0.0027 (7)	0.0118 (7)	0.0024 (7)
C24	0.0242 (9)	0.0211 (9)	0.0423 (9)	−0.0001 (7)	0.0163 (7)	0.0043 (7)
C25	0.0210 (8)	0.0211 (9)	0.0350 (9)	0.0009 (7)	0.0056 (7)	−0.0028 (7)
C26	0.0177 (8)	0.0197 (9)	0.0248 (7)	0.0056 (6)	0.0044 (6)	0.0010 (6)
C27	0.0298 (9)	0.0235 (9)	0.0263 (8)	0.0047 (7)	0.0062 (7)	−0.0050 (7)
C28	0.0363 (10)	0.0305 (10)	0.0214 (8)	0.0076 (7)	0.0111 (7)	−0.0020 (7)
C29	0.0284 (9)	0.0239 (9)	0.0262 (8)	0.0035 (7)	0.0146 (7)	0.0021 (7)
C30	0.0215 (8)	0.0231 (9)	0.0243 (7)	0.0002 (6)	0.0097 (7)	0.0034 (6)
C31	0.0235 (8)	0.0235 (9)	0.0292 (8)	0.0051 (7)	0.0061 (7)	−0.0004 (7)
C32	0.0301 (10)	0.0687 (14)	0.0294 (9)	0.0167 (9)	0.0133 (8)	0.0060 (9)
C33	0.0287 (9)	0.0394 (11)	0.0402 (10)	−0.0052 (8)	0.0116 (8)	−0.0022 (8)

### Geometric parameters (Å, °)

**Table:**

Cl1—C16	1.7469 (15)	C15—H15	0.9300
O1—C2	1.3761 (17)	C16—C17	1.375 (2)
O1—C1	1.4268 (18)	C17—C18	1.395 (2)
O2—C19	1.4299 (17)	C17—H17	0.9300
O2—H2	0.8200	C18—H18	0.9300
N1—C32	1.4576 (19)	C19—C20	1.546 (2)
N1—C33	1.463 (2)	C19—C30	1.548 (2)
N1—C31	1.476 (2)	C20—C29	1.379 (2)
C1—H1A	0.9600	C20—C21	1.447 (2)
C1—H1B	0.9600	C21—C22	1.4256 (19)
C1—H1C	0.9600	C21—C26	1.437 (2)
C2—C3	1.367 (2)	C22—C23	1.369 (2)
C2—C11	1.431 (2)	C22—H22	0.9300
C3—C4	1.421 (2)	C23—C24	1.402 (2)
C3—H3	0.9300	C23—H23	0.9300
C4—C5	1.417 (2)	C24—C25	1.357 (2)
C4—C9	1.421 (2)	C24—H24	0.9300
C5—C6	1.364 (2)	C25—C26	1.420 (2)
C5—H5	0.9300	C25—H25	0.9300
C6—C7	1.404 (2)	C26—C27	1.415 (2)
C6—H6	0.9300	C27—C28	1.357 (2)
C7—C8	1.368 (2)	C27—H27	0.9300
C7—H7	0.9300	C28—C29	1.402 (2)
C8—C9	1.418 (2)	C28—H28	0.9300
C8—H8	0.9300	C29—H29	0.9300
C9—C10	1.415 (2)	C30—C31	1.526 (2)
C10—C11	1.374 (2)	C30—H30A	0.9700
C10—H10	0.9300	C30—H30B	0.9700
C11—C12	1.517 (2)	C31—H31A	0.9700
C12—C13	1.532 (2)	C31—H31B	0.9700
C12—C19	1.567 (2)	C32—H32A	0.9600
C12—H12	0.9800	C32—H32B	0.9600
C13—C18	1.3881 (19)	C32—H32C	0.9600
C13—C14	1.392 (2)	C33—H33A	0.9600
C14—C15	1.383 (2)	C33—H33B	0.9600
C14—H14	0.9300	C33—H33C	0.9600
C15—C16	1.386 (2)		
			
C2—O1—C1	117.08 (12)	C13—C18—H18	119.1
C19—O2—H2	109.5	C17—C18—H18	119.1
C32—N1—C33	109.34 (13)	O2—C19—C20	109.47 (11)
C32—N1—C31	111.10 (14)	O2—C19—C30	108.55 (12)
C33—N1—C31	111.54 (12)	C20—C19—C30	110.76 (12)
O1—C1—H1A	109.5	O2—C19—C12	107.27 (11)
O1—C1—H1B	109.5	C20—C19—C12	110.87 (12)
H1A—C1—H1B	109.5	C30—C19—C12	109.82 (11)
O1—C1—H1C	109.5	C29—C20—C21	117.70 (14)
H1A—C1—H1C	109.5	C29—C20—C19	118.17 (13)
H1B—C1—H1C	109.5	C21—C20—C19	124.12 (12)
C3—C2—O1	123.29 (13)	C22—C21—C26	116.03 (13)
C3—C2—C11	121.53 (13)	C22—C21—C20	125.40 (13)
O1—C2—C11	115.18 (12)	C26—C21—C20	118.56 (13)
C2—C3—C4	120.75 (13)	C23—C22—C21	122.18 (14)
C2—C3—H3	119.6	C23—C22—H22	118.9
C4—C3—H3	119.6	C21—C22—H22	118.9
C5—C4—C9	119.03 (13)	C22—C23—C24	120.99 (14)
C5—C4—C3	122.48 (14)	C22—C23—H23	119.5
C9—C4—C3	118.46 (13)	C24—C23—H23	119.5
C6—C5—C4	120.84 (14)	C25—C24—C23	119.16 (15)
C6—C5—H5	119.6	C25—C24—H24	120.4
C4—C5—H5	119.6	C23—C24—H24	120.4
C5—C6—C7	120.38 (14)	C24—C25—C26	121.81 (15)
C5—C6—H6	119.8	C24—C25—H25	119.1
C7—C6—H6	119.8	C26—C25—H25	119.1
C8—C7—C6	120.28 (15)	C27—C26—C25	119.96 (14)
C8—C7—H7	119.9	C27—C26—C21	120.26 (14)
C6—C7—H7	119.9	C25—C26—C21	119.78 (13)
C7—C8—C9	121.03 (15)	C28—C27—C26	119.90 (15)
C7—C8—H8	119.5	C28—C27—H27	120.0
C9—C8—H8	119.5	C26—C27—H27	120.0
C10—C9—C8	122.33 (14)	C27—C28—C29	120.60 (14)
C10—C9—C4	119.22 (13)	C27—C28—H28	119.7
C8—C9—C4	118.43 (13)	C29—C28—H28	119.7
C11—C10—C9	122.17 (14)	C20—C29—C28	122.92 (14)
C11—C10—H10	118.9	C20—C29—H29	118.5
C9—C10—H10	118.9	C28—C29—H29	118.5
C10—C11—C2	117.87 (13)	C31—C30—C19	114.37 (12)
C10—C11—C12	123.90 (13)	C31—C30—H30A	108.7
C2—C11—C12	118.22 (12)	C19—C30—H30A	108.7
C11—C12—C13	111.65 (12)	C31—C30—H30B	108.7
C11—C12—C19	114.39 (12)	C19—C30—H30B	108.7
C13—C12—C19	113.64 (11)	H30A—C30—H30B	107.6
C11—C12—H12	105.4	N1—C31—C30	111.82 (13)
C13—C12—H12	105.4	N1—C31—H31A	109.3
C19—C12—H12	105.4	C30—C31—H31A	109.3
C18—C13—C14	117.61 (14)	N1—C31—H31B	109.3
C18—C13—C12	119.65 (13)	C30—C31—H31B	109.3
C14—C13—C12	122.73 (12)	H31A—C31—H31B	107.9
C15—C14—C13	121.66 (13)	N1—C32—H32A	109.5
C15—C14—H14	119.2	N1—C32—H32B	109.5
C13—C14—H14	119.2	H32A—C32—H32B	109.5
C14—C15—C16	119.00 (14)	N1—C32—H32C	109.5
C14—C15—H15	120.5	H32A—C32—H32C	109.5
C16—C15—H15	120.5	H32B—C32—H32C	109.5
C17—C16—C15	121.13 (14)	N1—C33—H33A	109.5
C17—C16—Cl1	119.99 (11)	N1—C33—H33B	109.5
C15—C16—Cl1	118.87 (12)	H33A—C33—H33B	109.5
C16—C17—C18	118.69 (13)	N1—C33—H33C	109.5
C16—C17—H17	120.7	H33A—C33—H33C	109.5
C18—C17—H17	120.7	H33B—C33—H33C	109.5
C13—C18—C17	121.78 (14)		
			
C1—O1—C2—C3	−31.0 (2)	C12—C13—C18—C17	−178.43 (13)
C1—O1—C2—C11	149.34 (14)	C16—C17—C18—C13	0.7 (2)
O1—C2—C3—C4	179.86 (13)	C11—C12—C19—O2	−69.07 (15)
C11—C2—C3—C4	−0.5 (2)	C13—C12—C19—O2	60.76 (15)
C2—C3—C4—C5	−178.10 (14)	C11—C12—C19—C20	50.41 (16)
C2—C3—C4—C9	0.1 (2)	C13—C12—C19—C20	−179.75 (11)
C9—C4—C5—C6	0.0 (2)	C11—C12—C19—C30	173.14 (12)
C3—C4—C5—C6	178.22 (15)	C13—C12—C19—C30	−57.03 (15)
C4—C5—C6—C7	−0.2 (2)	O2—C19—C20—C29	−4.46 (18)
C5—C6—C7—C8	0.6 (2)	C30—C19—C20—C29	115.19 (14)
C6—C7—C8—C9	−0.7 (2)	C12—C19—C20—C29	−122.62 (14)
C7—C8—C9—C10	−178.28 (15)	O2—C19—C20—C21	175.08 (12)
C7—C8—C9—C4	0.5 (2)	C30—C19—C20—C21	−65.26 (17)
C5—C4—C9—C10	178.70 (14)	C12—C19—C20—C21	56.92 (17)
C3—C4—C9—C10	0.4 (2)	C29—C20—C21—C22	−177.28 (14)
C5—C4—C9—C8	−0.1 (2)	C19—C20—C21—C22	3.2 (2)
C3—C4—C9—C8	−178.39 (14)	C29—C20—C21—C26	2.2 (2)
C8—C9—C10—C11	178.18 (14)	C19—C20—C21—C26	−177.31 (12)
C4—C9—C10—C11	−0.6 (2)	C26—C21—C22—C23	1.1 (2)
C9—C10—C11—C2	0.2 (2)	C20—C21—C22—C23	−179.36 (14)
C9—C10—C11—C12	−179.08 (13)	C21—C22—C23—C24	0.7 (2)
C3—C2—C11—C10	0.4 (2)	C22—C23—C24—C25	−1.3 (2)
O1—C2—C11—C10	−179.99 (13)	C23—C24—C25—C26	0.0 (2)
C3—C2—C11—C12	179.68 (13)	C24—C25—C26—C27	−177.40 (14)
O1—C2—C11—C12	−0.69 (19)	C24—C25—C26—C21	1.9 (2)
C10—C11—C12—C13	−87.94 (17)	C22—C21—C26—C27	176.94 (13)
C2—C11—C12—C13	92.80 (15)	C20—C21—C26—C27	−2.6 (2)
C10—C11—C12—C19	42.9 (2)	C22—C21—C26—C25	−2.3 (2)
C2—C11—C12—C19	−136.39 (13)	C20—C21—C26—C25	178.11 (13)
C11—C12—C13—C18	−111.46 (15)	C25—C26—C27—C28	−179.97 (14)
C19—C12—C13—C18	117.34 (14)	C21—C26—C27—C28	0.8 (2)
C11—C12—C13—C14	67.65 (17)	C26—C27—C28—C29	1.5 (2)
C19—C12—C13—C14	−63.55 (18)	C21—C20—C29—C28	−0.1 (2)
C18—C13—C14—C15	−3.3 (2)	C19—C20—C29—C28	179.50 (13)
C12—C13—C14—C15	177.58 (13)	C27—C28—C29—C20	−1.9 (2)
C13—C14—C15—C16	1.0 (2)	O2—C19—C30—C31	49.88 (17)
C14—C15—C16—C17	2.2 (2)	C20—C19—C30—C31	−70.33 (16)
C14—C15—C16—Cl1	−178.46 (11)	C12—C19—C30—C31	166.88 (13)
C15—C16—C17—C18	−3.1 (2)	C32—N1—C31—C30	164.04 (13)
Cl1—C16—C17—C18	177.63 (11)	C33—N1—C31—C30	−73.70 (16)
C14—C13—C18—C17	2.4 (2)	C19—C30—C31—N1	−68.12 (17)

### Hydrogen-bond geometry (Å, °)

**Table:**

*D*—H···*A*	*D*—H	H···*A*	*D*···*A*	*D*—H···*A*
O2—H2···N1	0.82	1.93	2.6995 (17)	157
